# Deaths during tuberculosis treatment among paediatric patients in a large tertiary hospital in Nigeria

**DOI:** 10.1371/journal.pone.0183270

**Published:** 2017-08-17

**Authors:** Aishatu L. Adamu, Muktar H. Aliyu, Najiba Aliyu Galadanci, Baba Maiyaki Musa, Muktar A. Gadanya, Auwalu U. Gajida, Taiwo G. Amole, Imam W. Bello, Safiya Gambo, Ibrahim Abubakar

**Affiliations:** 1 Department of Community Medicine, College of Health Sciences, Bayero University Kano, Kano, Nigeria; 2 Department of Community Medicine, Aminu Kano Teaching Hospital, Kano, Nigeria; 3 Department of Health Policy, Vanderbilt University School of Medicine, Nashville, TN, United States of America; 4 Vanderbilt Institute of Global Health, Nashville, TN, United States of America; 5 Department of Haematology, Aminu Kano Teaching Hospital, Kano, Nigeria; 6 Department of Medicine, College of Health Sciences, Bayero University Kano, Kano, Nigeria; 7 Department of Public Health and Disease Control, Kano State Ministry of Health, Nigeria; 8 Department of Paediatrics, Murtala Mohammed Specialist Hospital, Kano, Nigeria; 9 Institute for Global Health, University College London, London, United Kingdom; McGill University, CANADA

## Abstract

**Background:**

Despite availability of effective cure, tuberculosis (TB) remains a leading cause of death in children. In many high-burden countries, childhood TB is underdiagnosed and underreported, and care is often accessed too late, resulting in adverse treatment outcomes. In this study, we examined the time to death and its associated factors among a cohort of children that commenced TB treatment in a large treatment centre in northern Nigeria.

**Methods:**

This is a retrospective cohort study of children that started TB treatment between 2010 and 2014. We determined mortality rates per 100 person-months of treatment, as well as across treatment and calendar periods. We used Cox proportional hazards regression to determine adjusted hazard ratios (aHR) for factors associated with mortality.

**Results:**

Among 299 children with a median age 4 years and HIV prevalence of 33.4%; 85 (28.4%) died after 1,383 months of follow-up. Overall mortality rate was 6.1 per 100 person-months. Deaths occurred early during treatment and declined from 42.4 per 100 person-months in the 1^st^ week of treatment to 2.2 per 100 person-months after at the 3^rd^ month of treatment. Mortality was highest between October to December period (9.1 per 100 pm) and lowest between July and September (2.8 per 100 pm). Risk factors for mortality included previous TB treatment (aHR 2.04:95%CI;1.09–3.84); HIV infection (aHR 1.66:95%CI;1.02–2.71), having either extra-pulmonary disease (aHR 2.21:95%CI;1.26–3.89) or both pulmonary and extrapulmonary disease (aHR 3.03:95%CI;1.70–5.40).

**Conclusions:**

Mortality was high and occurred early during treatment in this cohort, likely indicative of poor access to prompt TB diagnosis and treatment. A redoubling of efforts at improving universal health coverage are required to achieve the End TB Strategy target of zero deaths from TB.

## Introduction

Tuberculosis (TB) is a major and often unrecognised cause of morbidity and mortality in children.[1] With an estimated 1 million new TB cases and over 200,000 deaths worldwide, children accounted for 10% of new infections and 12.5% of deaths in 2015.[2] Childhood TB represents recent and ongoing transmission and a potential reservoir from which the epidemic can continue in the future. TB in children presents with particular challenges including difficulties with bacteriologic confirmation, as sputum samples are often unobtainable with consequent underreporting.[3–5] Furthermore older children, with adult-type disease contribute to transmission.

Reducing mortality is an important target for TB control.[6] However, assessment of TB mortality is challenging in many high TB-burden countries due to poor or absent vital registration systems. The World Health Organization (WHO) recommends the use of deaths among persons with TB (case fatality) as an alternative approach to estimating TB-associated mortality.[2] Case fatality also assesses inequities in access to TB care.

In Nigeria, there were 586,000 new TB cases in 2015 with high levels of suboptimal outcomes; the lowest TB treatment coverage (15%) and the highest case fatality (43%) among the 30 high TB-burden countries.[2] Outcomes in children may be worse in view of the poor access to general health care. Based on national guidelines, TB cases as well as treatment outcomes in children should be notified to the TB programme. However, due to poor reporting, current data may not accurately reflect outcomes especially in subgroups where diagnosis may be more challenging and risk of death higher. In this study, we analysed data from a large tertiary hospital to examine levels of, and factors associated with, mortality among a cohort of children that commenced TB treatment.

## Materials and methods

### Study design and data sources

This was a retrospective cohort study with data obtained from clinic-based registers for patients at the Aminu Kano Teaching Hospital (AKTH). AKTH is a large tertiary hospital providing specialist services to a large catchment area comprising Kano metropolis and neighbouring states and general medical services to the local population. Data on age, gender, urban or rural residence, HIV status, disease site, prior TB treatment and treatment outcomes were obtained from the registers.

### Study population

Patients that enrolled in the clinic and commenced treatment between January 2010 and December 2014 and were less than 15 years of age were included in this analysis. Patients come from a variety of sources and they include both out-patients and in-patients. These sources comprise patients from other clinics from within the hospital including the paediatric out-patient clinic and HIV clinic; patients referred from other clinics and hospitals within and outside Kano; in-patients from the paediatric wards within the hospital. In our data, 83(27.8%) were in-patients. Information on all subjects included in our analysis were from the TB clinic-based registers because all patients with a TB diagnosis are registered in the clinic irrespective of their source. For in-patients, the managing team collects the patient information and comes to the register the patient in the clinic. Diagnosis and treatment were according to the Nigerian National TB and Leprosy Control Programme guidelines. Diagnosis of TB is largely based on a composite score comprising a combination of clinical and laboratory findings: unexplained fever, chest radiography abnormalities, history of close contact with a smear-positive pulmonary TB patient, undernutrition (assessed using weight-for-height), poor or non-response to antibiotics, and tuberculin skin test (TST) induration size. Patients presenting with cough lasting for 2 weeks or more had sputum sent to the TB laboratory for bacteriologic confirmation through smear microscopic examination. Sputum was obtained either by expectoration (from children that could produce sputum) or gastric lavage. Chest X-rays were also obtained for younger children and those unable to produce sputum if pulmonary tuberculosis was suspected. Diagnosis of extra pulmonary tuberculosis (EPTB) was sometimes based on fine needle aspiration cytology, histopathological examination, biochemical and microscopic analyses of cerebrospinal/pleural/ascitic fluids.

Treatment for new cases of TB comprised two phases: a 2-month intensive phase with Rifampicin, Isoniazid, Ethambutol and Pyrazinamide; and a 4 or 6-month continuation phase with Rifampicin and Isoniazid. Prior to 2014, patients with history of TB treatment had 3-month intensive phase with Streptomycin injection added to the regimen. Paediatric drugs are generally provided free under the State TB Control Programme. However, occasional stock-outs occur and in such cases, appropriate doses are reconstituted from loose adult tablets in the hospital drug manufacturing unit or patients are asked to obtain their drugs from private pharmaceutical outlets.

### Treatment outcome definitions

Treatment outcomes were defined using the WHO TB treatment outcome definitions.[7] Death included loss of life from any cause after treatment commenced. Cure was defined as smear- or culture-negative status in the last month of treatment in a patient who was bacteriologically confirmed at treatment onset. Treatment completion was defined as any patient who completed treatment without evidence of failure and with no record of a negative smear or culture result in the last month of treatment, while treatment failure was defined as being sputum or culture-positive at the 5^th^ month of treatment. Treatment interruption for 2 consecutive months or more was characterized as “loss to follow up”. Successful treatment included patients categorised as “cured” or “treatment completed”.

### Statistical analysis

All analyses were conducted using Stata 14 (Stata Corp, College Station, TX, USA). Cohort entry was defined as the date of treatment initiation. All subjects were followed up until the first to occur from the following dates: death; treatment failure; loss to follow-up; and treatment completion. To determine mortality rates, follow-up time was set as time from treatment initiation and expanded into person-months (pm). Mortality rates per 100pm were estimated. Cox proportional hazard modelling was used to estimate hazard ratios (HR) for mortality. In addition to age, we adjusted for covariates that were associated with mortality at univariable analysis with p<0.2 (gender, residence, HIV status, disease site, prior TB treatment and calendar year).

We also determined timing of death in relation to treatment duration and calendar period. To examine death in relation to treatment duration, follow-up time was split into weekly/monthly intervals; and into the treatment phases (intensive and continuation). To examine death in relation to calendar period, follow up time was set as calendar year and each year was split into quarters.

### Ethics

Ethical approval was obtained from the Research Ethics Committee of AKTH (NHREC/21/08/2008/AKTH/EC/1562). Patient consent was not required by the ethics committee since data were obtained retrospectively from patients’ records.

## Results

Between 2010 and 2014, 312 children commenced TB treatment with 299 (95.8%) children included in the analysis as 13 were excluded due to incomplete information on either treatment outcome or date of treatment initiation. Children represented 21% of the 1,796 patients enrolled in the clinic over the study period. The characteristics of the study population are summarised in Table 1. There were more boys than girls with a male to female ratio of 1.34:1. The age of participants ranged from 3 months to 14 years with a median (IQR) of 4 years (1.5–9). One-third (33.4%) of the children were HIV-positive. Pulmonary disease was the most common disease presentation (63.9%), while central nervous system disease accounted for 5.7% of all cases.

**Table 1:** Description of study cohort and mortality rates across explanatory variables, TB clinic, Aminu Kano Teaching Hospital, Kano, Nigeria.

Variable		Total	Number of deaths (%)	Person-months	Mortality rate per 100pm (95%CI)
**Age group (years)**					
	<5	152	44(29.0)	695.6	6.3(4.7–8.5)
	5–9	80	22(27.5)	377.3	5.8(3.8–8.9)
	≥ 10	67	19(28.4)	310.4	6.1(3.9–9.6)
**Gender**					
	Male	171	53(31.0)	751	7.1(5.4–9.2)
	Female	128	32(25.0)	632.3	5.1(3.6–7.2)
**Residence**					
	Urban	196	47(24.0)	956.5	4.9(3.7–6.5)
	Rural	103	38(36.9)	426.8	8.9(6.5–12.2)
**HIV status**					
	Negative	189	43(21.6)	1011	4.3(3.2–5.7)
	Positive	100	42(42.0)	372.3	11.3(8.3–15.3)
	Unknown	10	2(20.0)	46.5	4.3(1.1–17.2)
**Previous TB treatment**					
	No	267	71(26.6)	1256.1	5.7(4.5–7.1)
	Yes	32	14(43.8)	127.3	11.0(6.5–18.6)
**Disease site**					
	Pulmonary	191	40(20.9)	910.3	4.4(3.2–6.0)
	Extrapulmonary	66	24(36.4)	306.9	7.8(5.2–11.7)
	Both	42	21(50.0)	166.2	12.6(8.2–19.4)
**Calendar year**					
	2010–2011	58	31(53.5)	154.1	20.1(14.1–28.6)
	2012	64	19(29.7)	283.9	6.7(4.3–10.5)
	2013	106	19(17.9)	545.7	3.5(2.2–5.5)
	2014	71	16(22.5)	399.6	4.0(2.5–6.5)

### Treatment outcome

Distribution of treatment outcome over the study period is summarised in Table 2. About half of the children (52.5%) had a successful treatment outcome. Overall, among children with unsuccessful treatment outcomes, 85 (59.9%) died, 1 (0.7%) failed treatment and 56 (39.4%) were lost to follow-up during treatment. The proportion of patients with successful treatment outcome progressively increased from 17% in the 2010/2011 period to 65% in 2014. The proportion lost to follow-up also declined from 29.3% in 2010/2011 to 11% in 2014.

**Table 2:** Treatment outcomes of children over study period, TB clinic, Aminu Kano Teaching Hospital, Kano, Nigeria.

	Calendar year				
Treatment outcome	2010–2011 n (%)	2012 n (%)	2013 n (%)	2014 n (%)	Total n (%)
Successful treatment	10(17.2)	30(46.9)	71(67.0)	46(64.8)	157(52.5)
Died	31(53.5)	19(29.7)	19(17.9)	19(22.5)	85(28.4)
Lost to follow-up	17(29.3)	15(23.4)	16(15.1)	8(11.3)	56(18.7)
Failed treatment	0(0.0)	0(0.0)	0(0.0)	1(1.4)	1(0.3)

### Mortality rates

There were 85 deaths during a total follow-up time of 1383.3 months, with an overall mortality rate of 6.1 per 100 person-months (95% CI: 5.0–7.6). Crude mortality rates across patient characteristics are shown in Table 1. Mortality rates were similar across all age groups, but slightly higher among boys (7.1/100pm: 95% CI 5.4–9.2) than girls (5.1/100pm; 95% CI: 3.6–7.2). HIV-positive children also had a higher mortality (11.3/100pm; 95% CI: 8.3–15.3) than children who were HIV-negative (4.3/100pm; 95%CI: 3.2–5.7) or had unknown HIV status (4.3/100pm; 95% CI: 1.1–17.2). Mortality declined between 2010 and 2014. Crude analysis revealed that compared to urban-dwelling children, those living in rural areas were 68% more likely to die (95% CI: 1.09–2.58).

After adjusting for age, gender, residence, HIV status, history of prior TB treatment, disease site and calendar period; being HIV-positive (aHR 1.66;95%CI:1.02–2.71); history of prior TB treatment (aHR 2.04;95%CI:1.09–3.84); and having either extrapulmonary (aHR 2.21;95%CI:1.26–3.89) or both extrapulmonary and pulmonary disease (aHR 3.03;95%CI:1.70–5.40) were found to be independent risk factors for mortality in the study population (Table 3).

**Table 3:** Univariable and multivariable analyses of risk factors for mortality among children, TB clinic, Aminu Kano Teaching Hospital, Kano, Nigeria.

Variable	Number of deaths (%)	Crude HR (95% CI)	P value (LRT)^1^	Adjusted HR (95% CI)^2^	P value (LRT)^1^
**Age**					
<5	44(29.0)	0.98(0.57–1.68)		1.25(0.70–2.21)	
5–9years	22(27.5)	0.94(0.51–1.74)		1.01(0.53–1.09)	
10years+	19(28.4)	1	0.98	1	0.65
**Gender**					
Male	53(31.0)	1		1	
Female	32(25.0)	0.74(0.47–1.15)	0.18	0.65(0.41–1.03)	0.07
**Residence**					
Urban	47(24.0)	1		1	
Rural	38(36.9)	1.68(1.09–2.58)	0.02	1.47(0.94–2.29)	0.1
**HIV status**					
Negative	43(21.6)	1		1	
Positive	42(42.0)	2.40(1.57–3.70)	0.0001	1.66(1.02–2.71)	
Unknown	2(20.0)	0.96(0.23–3.98)		0.92(0.21–3.96)	0.11
**Previous TB treatment**					
No	71(26.6)	1		1	
Yes	14(43.8)	1.83(1.03–3.26)	0.05	2.04(1.09–3.84)	0.04
**Disease site**					
Pulmonary	40(20.9)	1		1	
Extrapulmonary	24(36.4)	1.78(1.07–2.96)		2.21(1.26–3.89)	
Both	21(50.0)	2.71(1.60–4.60)	0.001	3.03(1.70–5.40)	0.0004
**Calendar year**					
2010–2011	31(53.5)	1			
2012	19(29.7)	0.38(0.22–0.68)		0.52(0.27–1.00)	
2013	19(17.9)	0.22(0.12–0.38)		0.20(0.10–0.38)	
2014	16(22.5)	0.25(0.14–0.470	<0.0001	0.25(0.13–0.50)	<0.0001

^1^ Likelihood ratio test

^2^ Adjusted for all variables in the table

### Timing of deaths

The median survival time for patients that died was 14 days (IQR: 6–54 days). About a third (35.3%) of the deaths occurred within the 1st week of treatment with a crude mortality of 42.4 (95% CI: 29.6–60.6) per 100 person-months in the 1^st^ week of treatment. Mortality steadily declined over the treatment period to 2.2 (95% CI: 1.4–3.6) per 100 person-months among children that had been on treatment for 4 months or more. When treatment period was analysed by treatment phases, risk of mortality was nearly 8 times higher during intensive phase compared to the continuation phase. This increased risk of death in the earlier periods of treatment remained after adjusting for age, gender, residence, HIV status, history of prior TB treatment, and disease site.

Analysis of timing death across calendar period showed that mortality was highest between October and December period (9.1/100pm; 95% CI: 6.3–13.3), with more than twice the risk of death compared to January to March period, on both crude and adjusted analyses (Table 4).

**Table 4:** Timing of deaths among children on TB treatment, TB clinic, Aminu Kano Teaching Hospital, Kano, Nigeria.

Time since treatment onset	Number of deaths	Proportionate mortality (%)	Person-months	Mortality rate per 100pm (95%CI)	Crude HR (95% CI)	Adjusted HR (95% CI)^1^
**Time since treatment onset**						
1st week	30	35.3	70.8	42.4(29.6–60.6)	18.34(8.65–38.92)	17.66(8.10–38.49)
2nd week	18	21.2	63.1	28.5(18.0–45.3)	21.17(9.2–48.47)	23.18(9.68–55.52)
3rd week	5	5.9	58.3	8.6(3.6–20.6)	4.93(1.58–15.37)	4.80(1.49–15.46)
Fourth week	4	6.2	56.7	7.1(2.6–18.8)	4.30(1.32–13.96)	3.57(1.05–12.11)
2nd month	8	9.4	212.3	3.8(1.9–7.5)	2.37(0.92–6.13)	2.19(0.83–5.79)
3rd month	4	4.7	199.6	2.0(0.8–5.3)	1.04(0.92–6.13)	0.92(0.28–2.99)
4th month+	16	18.8	722.5	2.2(1.4–3.6)	1	1
Total	85		1383.3	6.1(5.0–7.6)		
**Treatment phase**						
Intensive phase	65	76.5	461.2	14.1(11.1–18.0)	7.86(4.21–14.00)	7.89(4.38–14.20)
Continuation phase	20	23.5	922.1	2.2(1.4–3.4)	1	1
**Calendar period(season)**						
Jan-Mar	32	37.6	660.8	4.8(3.4–6.8)	1	1
Apr-Jun	10	11.8	147.5	6.8(3.6–12.6)	0.78(0.37–1.56)	0.73(0.36–1.52)
Jul-Sept	16	18.8	279.9	2.8(3.5–9.3)	0.87(0.48–1.60)	0.83(0.45–1.55)
Oct-Dec	27	31.8	295.1	9.1(6.3–13.3)	2.07(1.23–3.46)	2.18(1.29–3.70)

^1^ adjusted for age, place of residence, gender, HIV status, disease site, and history of previous TB treatment

## Discussion

This study demonstrates unacceptably poor outcomes with high mortality among children during TB treatment in this large urban centre. Overall, over a quarter of the children that started TB treatment died, and these deaths occurred early during treatment, with over three-quarters of the deaths occurring in the intensive phase of treatment. We also show variation in death rates over time. Deaths declined steadily over the study period suggesting improvement in recent years, but varied within each study year. Our results also indicate that disease site and history of prior TB treatment were independent risk factors for mortality.

Compared to other single-hospital studies, reported deaths were still lower than our findings. In a referral hospital Peru, analysis of 25-year data of hospitalised children with TB reported 11% mortality.[8] In Ethiopia, analysis of data from a rural hospital over 10 years reported 3.9% deaths.[9] In the Democratic Republic of Congo, a 5-year retrospective analysis of TB data from a tertiary-level health facility, reported only 1.4% deaths.[10] However, our study population comprised a relatively younger (median age– 4 years) with a much higher HIV prevalence.

As expected mortality during treatment is higher than reported in many studies using programmatic data from other high TB-burden populations.[11–15] In Lagos, southern Nigeria, analysis of state-wide TB data showed that 6% of children died while on treatment.[15] In Benin, a 2-year retrospective study reported 9% mortality.[11] In southern Ethiopia, analysis of 5-year district-wide treatment outcome reported that 3% of the children died.[12] In Malawi, a nation-wide survey of programme data in 1998 reported that 17% of the children died; [13] while analysis of 3-year (2011–2013) programme data from treatment centres in central Malawi reported 9.5% deaths.[14] Likely inclusion of more severe cases with higher mortality risk, would at least in part explain the difference between these studies and our findings.

The high and early mortality during treatment in this study could be attributable to many factors. Delay in diagnosis could result in disease progression because of the difficulties in diagnosis of childhood TB, especially if cough is absent, or sputum cannot be made for bacteriological examination, or in EPTB.[16] Due to these diagnostic challenges, lower-cadre health care workers at primary care level where the majority of TB care is provided may fail to suspect TB in children or may not have access to diagnostic equipment, such as radiographs.[17] Because a number of patients were referred from other lower-level hospitals, it is possible that lower cadre health care workers at this levels may face greater challenges in diagnosis of TB in children and this may have contributed to overall delays in diagnosis and treatment initiation. Frequent drug stock-outs may also cause delayed treatment; and compromised adherence; resulting in inadequate dosing from reconstituted adult drugs; or risked ingestion of adulterated or expired drugs.[17] Treatment adherence may be poor in children and worsened by epileptic drug supply, poor tolerance, and socio-economic factors.[18] Though microscopy and drugs (when available) are provided free by the TB programme, transportation and other diagnostic tests are out-of-pocket expenses which may negatively affect adherence and treatment outcome.[19] High HIV-burden (a third were co-infected) in this cohort could also have contributed to high mortality from delayed diagnosis; co-morbidities from other opportunistic infections; and drug interactions potentially compromising adherence or effectiveness of anti-TB drugs.[20–22] Drug-susceptibility testing only became available in 2014 in the centre, therefore, undiagnosed drug resistant TB may also have contributed to high mortality, especially with previous history of TB treatment being a strong predictor of death.

The relatively high HIV co-infection in our cohort (33.4%) is similar to levels reported from Lagos (29%), another highly populous state in Nigeria. [15] [23] Other states in the country reported much lower levels of coinfection. In Zaria, northern Nigeria, a hospital-based study found coinfection levels of 9%. [24] A state-wide study in south-west Nigeria also showed coinfection of 11%. [25] In AKTH coinfection among HIV-infected adults was 10.5% [26] and 40% among TB patients [27]. In southern Africa, coinfection levels are higher ranging from 42% to 56% [28–32]. While in other parts of sub-Saharan Africa coinfection levels are lower than our findings. [33] [34] Though HIV prevalence in Kano state is relatively low, the high level of coinfection in this cohort may be attributed to the fact the study site is one of the largest HIV service providers in the state.

Though mortality did not vary with age, the relative young age of this cohort could have accounted for the overall high mortality. Diagnosis is more challenging in younger children as clinical features are not distinct to TB, but largely similar to other prevalent childhood diseases.[35] An immature immune system in younger children results in disseminated disease, which further complicates diagnosis, [15] [16] and increases the risk of disease progression.[36] Additionally, higher prevalence of other co-morbidities which are prevalent in this population, such as severe undernutrition, pneumonia and undiagnosed HIV infection contribute to early deaths.[16]

Other studies have reported timing of deaths during treatment. In a 10-year hospital-based study in Ethiopia, the mean survival time was low (10 days) in children despite overall low mortality (3.9%).[9] In a retrospective analysis of data from two referral centres in South Africa, median survival time was 112 days among hospitalised HIV-positive patients with high levels of poor treatment response.[37] In India, a retrospective review of treatment outcome showed consistent deaths throughout the treatment period, which is attributed to underestimation of early deaths due to high default and poor treatment adherence in the cohort.[38]

The temporal trend in improvement of treatment success and reduction of mortality over the study period (2010 to 2014) in our study could be attributed to expansion of TB services including availability of paediatric drugs; expansion of HIV services including ART; and likely maturity of the programme within the hospital and in the country at large.

Seasonal variation in mortality observed may reflect underlying burden of other childhood diseases such as respiratory tract infections as well as TB incidence which in turn can increase the risk of death.[39, 40] In South Africa, an increase in hospitalisation from PTB and pneumococcal disease after the influenza season was reported.[41] The presence of such co-morbidities may also have led to overdiagnosis of TB and wrong treatment; and contributed to high mortality in our cohort.[42] Pulmonary disease was the most common presentation in this cohort, and majority of participants (77.9%) had either PTB alone or in combination with EPTB. It is likely that other respiratory tract infections could have contributed to mortality in our cohort.

Major strengths of this study include the availability of dates to allow time-to-event analysis, and inclusion of patients that access TB drugs outside the TB programme.

This study relied on retrospective clinic registers and, therefore, includes only data reported in the registers or patient treatment cards. Other important clinical data such as nutritional status, use of isoniazid prophylaxis, BCG vaccination, anti-retroviral treatment, CD4 cell count were either not available or missing from the registers. Socio-economic data such as family income, parental education, which influence treatment-seeking are also unavailable from these registers. The representativeness of our findings may be limited to similar tertiary-level TB treatment centres. However, due to the diversity of the patients and the fact that the diagnosis of smear-negative TB and EPTB in children often relies on paediatricians or pulmonologists located at higher-level hospitals, our findings represent an important sub-group with increased risk of poor outcome that may not be so apparent from programme-level data. Our findings are likely to underestimate overall mortality, as we do not account for deaths before diagnosis or treatment initiation; and deaths after treatment from relapse, treatment failure or other TB sequelae.[37, 43] Though efforts are made to contact patients lost to follow-up through the contact number of guardian provided to the clinic on enrolment before treatment outcome is registered, it is possible that among patients that could not be reached, a number of them could have died and this may have resulted in underestimation of mortality in this cohort. Conversely, it is possible that some deaths included in our analysis may be unrelated to TB. However, because vital registration is poor, analysis of TB treatment cohort such as this one provides an insight into the quality of TB care in this subpopulation.

## Conclusion

The high and early mortality of children on TB treatment points to significant inequities in access to TB care. Diagnosis and treatment are delayed; and adherence may be poor, and these lead to adverse treatment outcomes. These delays may be reflective of overall poor health care access among this population. Temporal variations in mortality also suggest that background incidence of other common childhood infections may be contributing to deaths in children. Our findings point towards the importance of the End TB Strategy’s targets of universal health coverage and social protection for ending TB by 2035.[44]
